# Stability data of extemporaneous suspensions of hydroxychloroquine sulphate in oral liquid bases after tablet manipulation

**DOI:** 10.1016/j.dib.2020.106575

**Published:** 2020-11-30

**Authors:** Mara Perrone, Valentino Laquintana, Angela Assunta Lopedota, Annalisa Cutrignelli, Antonio Lopalco, Massimo Franco, Antonio Pepe, Sergio Fontana, Nunzio Denora

**Affiliations:** a Centro Studi e Ricerche “Dr. S. Fontana 1900-1982”, Farmalabor s.r.l., Via Piano S. Giovanni, 47, 76012 Canosa di Puglia (BT), Italy; b Department of Pharmacy – Pharmaceutical Sciences, University of Bari “Aldo Moro”, Via E. Orabona, 4, 70125 Bari, Italy

**Keywords:** Hydroxychloroquine sulphate, Plaquenil, Galenic formulation, Liquid formulation, Turbiscan lab expert, Extemporaneous suspension, Oral liquid bases

## Abstract

Hydroxychloroquine is a well-known anti-malarial and anti-rheumatic drug that has garnered recently unprecedented attention as potential therapeutic agent against virus infections. Hydroxychloroquine sulphate is authorized in the EU as film-coated tablets (i.e. Plaquenil®), which cannot be administered to non-cooperative patients, such as those in intensive care units or, more in general, unable to swallow solid dosage forms. Therefore, the hospital pharmacist must manipulate the solid dosage form for the preparation of suspension, even if it can strongly affect the product quality. In this scenario, it is crucial to offer useful information and advice to assist hospital pharmacists in their activity. The data presented in this article suggest that extemporaneous suspensions of hydroxychloroquine sulphate in oral liquid bases after tablet manipulation are stable for at least 30 days.

## Specifications Table

**Table:** 

Subject	Pharmacology, Toxicology and Pharmaceutical Science
Specific subject area	Pharmaceutical Science
Type of data	Table Figure Text
How data were acquired	High-Performance Liquid Chromatography (HPLC) Turbiscan lab expert optical analyser
Data format	Raw Analysed
Parameters for data collection	Data on hydroxichloroquine sulphate stability in suspension through one month from preparation after storage at 4 °C and at room temperature (RT)
Description of data collection	The drug suspensions were prepared in the laboratory of the Study and Research center “Dr. S. Fontana 1900–1982″ manipulating commercial tablets of hydroxichloroquine sulphate (i.e. Plaquenil®) and using two oral vehicles (fast oral solution, named Puccini and fast oral solution - sugar free, named Beethoven). The suspensions were stored at 4 °C and RT for one month and at scheduled times the samples were collected and directly analysed by HPLC and by Turbiscan lab expert analysis.
Data source location	Bari and Canosa di Puglia (BT), Puglia, Italy
Data accessibility	Analysed data with the article. Figures, additional tables and raw data with supplementary data. http://dx.doi.org/10.17632/yznh3nzg8b.1

## Value of the Data

• The data demonstrate that hydroxychloroquine sulphate is chemically stable when a crushed tablet is suspended in specific vehicles and stored for one month at different temperatures. The data also afford indications on the physical and microbiological stabilities of extemporaneously prepared suspensions of hydroxychloroquine sulphate in selected vehicles after tablet manipulation.
• The data can support hospital pharmacists and clinicians in their activities aimed to cover the gaps generated by the absence of appropriated commercial liquid dosage forms.
• The data presented in this work are insights for additional investigations aimed on the development of new formulations dedicated to patients for which an oral liquid preparation is more appropriated than a solid one.

## 1 Data Description

Hydroxychloroquine sulphate is authorized in the EU as film-coated tablets (i.e. Plaquenil®), which cannot be administered to non-cooperative patients, such as those in intensive care units or, more in general, unable to swallow solid dosage forms. Thus, the role of pharmacists in the preparations of extemporaneous suspensions by manipulation of the authorized medicinal product is critical and must guarantee a final product without altering the efficacy and safety of the pharmacological treatment. Therefore, it is required that such compounding activities must be guided by the provisions of the Good Compounding Practice and by other available technical guidelines to assure the required quality and the stability of the preparation over time [1].

Regarding hydroxychloroquine sulphate, when an oral liquid formulation is more appropriated than the solid dosage form, the consolidated compounding activity of pharmacists consists on crushing the commercial tablet and in turn preparing a stable oral suspension.

Tables 1 and 2 reported the data on drug assay obtained storing extemporaneous suspensions of the powder obtained by the manipulation of the drug at RT and at 4 °C.

**Table 1:** Data on the chemical stability of hydroxychloroquine sulphate in fast oral solution “Puccini”-based extemporaneous suspensions when stored at RT or 4 °C for one month (30 days). Data are expressed as mean of tree analysis and standard deviation (SD).

Storage condition	Sampling times (days)	Hydroxychloroquine sulphate	
		Drug assay (mg/mL)	SD (mg/mL)
4 °C	0	30.02	0.27
	3	30.05	0.32
	7	30.10	0.18
	14	29.94	0.17
	21	29.90	0.07
	30	29.96	0.07
RT	0	30.05	0.18
	3	29.92	0.21
	7	29.91	0.42
	14	29.99	0.39
	21	30.06	0.06
	30	30.01	0.15

**Table 2:** Data on the chemical stability of hydroxychloroquine sulphate in fast oral solution – sugar free “Beethoven”-based extemporaneous suspensions when stored at RT or 4 °C for one month (30 days). Data are expressed as mean of tree analysis and standard deviation (SD).

Storage condition	Sampling times (days)	Hydroxychloroquine sulphate	
		Drug assay (mg/mL)	SD (mg/mL)
4 °C	0	30.01	0.16
	3	30.12	0.34
	7	30.05	0.15
	14	29.98	0.22
	21	29.93	0.18
	30	29.96	0.09
RT	0	30.04	0.23
	3	29.89	0.18
	7	29.84	0.34
	14	30.01	0.12
	21	29.99	0.17
	30	30.12	0.09

The data confirm that hydroxychloroquine is chemically stable in the suspensions over one month (30 days), regardless of the vehicle and the storage condition.

The physical stability of the suspensions was studied using Turbiscan technology [2]. In Fig. S1 (panels A and B), the backscattering profile (BS) of the extemporaneous suspension of hydroxychloroquine sulphate in fast oral solution “Puccini” studied at 30 °C shows after 1 h the presence of instability phenomena related both to migration and sedimentation with a ΔBS ≈ 40%. Instead, the extemporaneous suspension of hydroxychloroquine sulphate in fast oral solution – sugar free “Beethoven” appears stable with a ΔBS not significant (ΔBS < 10%) and a slight phenomenon of migration and sedimentation measured after 12 h of analysis (Fig. S1, panel C).

Fig. S2 (panel A and B) shows the variation of the Turbiscan Stability Index (TSI). A TSI value within 2 indicates the absence of destabilization phenomena, while unstable systems show TSI values higher than 2. The TSI data confirms the evident phenomenon of sedimentation of the tablet insoluble excipients in the vehicle “Puccini” with a TSI = 14 on the bottom of the tube (TSI = 11 overall) (Fig. S2, panel A). Instead, as reported in Fig. S2 (panel B), the TSI data recorder for fast oral solution – sugar free “Beethoven”-based extemporaneous suspension is very low (TSI= 0.32 on the bottom and TSI= 0.25 overall) confirming the high physical stability.

Table 3 reported the microbiological stability data of the extemporaneous suspensions of hydroxychloroquine sulphate in the two commercial vehicles. The data show that both formulations are microbiologically stable over one month (30 days).

**Table 3:** Data on the microbiological stability of extemporaneous suspensions of hydroxychloroquine sulphate in both fast oral solution “Puccini” and fast oral solution - sugar free “Beethoven” vehicles when stored at RT for one month (30 days).

Vehicle	Sampling time (days)	Microbiological assay	
		Total Microbial Count (cfu/g)*	Yeasts and Moulds (cfu/g)⁎⁎
fast oral solution “Puccini”	0	<10	0
	30	<10	0
fast oral solution - sugar free “Beethoven”	0	<10	0
	30	<10	0

⁎ Compliant limit: <1000.

⁎⁎ Compliant limit: <100.

## 2 Experimental Design, Materials and Methods

### 2.1 Materials

Plaquenil® 200 mg film coated tablets (Sanofi, EU): 200 mg hydroxychloroquine sulphate, equivalent to 155 mg hydroxychloroquine (base). Tablet excipients: lactose monohydrate, povidone, maize starch, magnesium stearate, opadry OY-l- 28,900 (hypromellose, macrogol 400, titanium dioxide, lactose monohydrate). Fast oral solution “Puccini” (composition: sucrose 54%; glycerol 5%; sorbitol 4%; citric acid 0.07%; sodium phosphate dibasic 0.09%; potassium sorbate 0.1%; highly purified water q.s. to 100% w/w) and fast oral solution - sugar free “Beethoven” (composition: glycerol 10%, sorbitol 9%, sucralose 0.1%, xanthan gum 1%, citric acid 0.09%, sodium citrate 0.09%, potassium sorbate 0.18%, highly purified water q.s. to 100% w/w) were gifted by Farmalabor srl. Hydroxychloroquine sulphate (Pharmaceutical Secondary Standard) and all other chemicals/solvents used in the study were either analytical grade or used without further purification.

### 2.2 Suspension preparation

Extemporaneous suspensions of hydroxychloroquine sulphate (30 mg/mL) were prepared by crushing commercial tablet of Plaquenil® in a mortar to obtain a fine and homogenous powder. The volume of vehicle needed to set up the suspension was measured with a graduated cylinder and transferred in a glass becker. The powder of Plaquenil® was added to each of the select bases, fast oral solution “Puccini” or fast oral solution - sugar free “Beethoven”, under continues magnetic stirring. A small quantity of the measured base was used to clean the mortar and pestle and subsequently added to the suspension reaching the final volume of 100 mL. The vehicle volume was set up to obtain a final suspension containing hydroxichloroquine sulphate at a concentration of 30 mg/mL. The suspensions were prepared three times, aliquoted in 100 mL amber glass bottles and stored at two storage temperatures (RT and 4 °C, three samples for each temperature) until analysis.

### 2.3 Chemical stability studies

Aliquots of suspension were collected, after shacking of the formulation, from each of the stored samples at time zero and after 3, 7, 14, 21 and 30 days. The collected aliquots were heated to RT, where necessary, and vigorously mixed for 5 s using a vortex and, then, sonicated until obtaining a homogeneous suspension. 0.1 mL of collected sample was diluted into 20 mL of methanol and was vigorously mixed for 5 s using a vortex before being analysed by HPLC.

### 2.4 HPLC method

HPLC analyses were performed with an Agilent 1260 Infinity Quaternary LC System equipped with an Agilent variable wavelength UV detector, a Rheodyne injector (Rheodyne, Model 7725i) equipped with a 20 µL loop and a OpenLAB CDS ChemStation software (Agilent, Santa Clara, CA). HPLC analyses were accomplished in reversed phase using a Zorbax C18 column (150 mm × 4 mm; 5 µm particles) as stationary phase. All analyses were performed in isocratic conditions. The injected volume was 20 µL. The flow rate was set at 0.8 mL/min and the column temperature at 40 °C. The column eluent was continuously monitored at 268 nm. The mobile phase was composed of a mixture of 50/50 v/v of solvent A: HPLC-grade water containing 10 mM ammonium acetate and 0.1% formic acid; solvent B: acetonitrile HPLC grade. Mobile phase solutions were filtered (cellulose regenerate, pore size 0.45 µm) and degassed before use. The amount of hydroxychloroquine in the collected samples was measured by the chromatograph peak area in relation to those obtained from the standards samples analysed under the same conditions. Chromatogram of control hydroxychloroquine sulphate and analyses’ raw data were described in Fig. S3 and Tables S1–2 in the supplementary materials.

### 2.5 Standard curves and linearity

Pure hydroxychloroquine sulphate was used to prepare a 5.0 mM stock solution using a water/DMSO 50/50 v/v mixture. HPLC-grade water was used to prepare serial dilutions of the stock solution. The lower limit of detection (LOD) and lower limit of quantification (LOQ) for hydroxychloroquine sulphate was evaluated on standard deviation (S.D.) of response and the slope of the calibration curve. S.D. of the response was estimated by calculating the S.D. of y-intercept of regression line and the slope was calculated from the calibration curve. The linearity of the method was demonstrated using seven different concentrations of hydroxychloroquine sulphate in a range between 0.488 µM and 1 mM. Each concentration peak area was recorded in triplicate, and taken average area from triplicate injections. The linearity was determined by least squares regression analysis. Linearity ranged between 0.488 μM and 1 mM (*R*^2^=0.9987), LOD was 0.065 μg/mL and the LOQ was 0.22 μg/mL.

### 2.6 Turbiscan stability analysis

Physical stability measurements of the suspensions were conducted using the Turbiscan Lab Expert (Formulaction, L'Union, France) optical analyser. Each sample (20 mL) was placed into cylindrical glass tubes (cell higher 40 mm) and subsequently analysed at 30 °C. Transmission (T) and backscattering (BS) intensity measurements were carried out using a pulsed near infrared LED at a wavelength of 880 nm for scheduled times (1 h for fast oral solution “Puccini”, 12 h for fast oral solution - sugar free “Beethoven”) and the outlines were analysed as a function of the height of the sample cell, acquiring every 40 µm, over time. Turbiscan profiles and analyses’ raw data were described in Figs. S1–3 and Tables S3–5 in the supplementary materials.

### 2.7 Microbiological stability

Microbial contamination is determined by the total viable aerobic count, which is the sum of the bacterial and the fungal counts. The tests allow quantitative enumeration of mesophilic bacteria and fungi that may grow under aerobic conditions. Plate count method was used for determination of total viable aerobic count. According to the Italian Pharmacopeia XII edition (IPhXII), the sample was prepared diluting 10 g or 10 mL of each product to be examined in buffered sodium chloride-peptone solution pH 7.0. Subsequently, a one in ten dilution was prepared for each sample. The acceptance limit for this assay is not more than 10^3^ bacteria and not more than 10^2^ fungus per g or mL of the preparation. Furthermore, according to IPhXII, the product complies with the test if no colonies are present or if the identification tests are negative.

## CRediT Author Statement

**Mara Perrone**: Data curation, Investigation, **Valentino Laquintana**: Investigation, Writing - Original Draft, **Angela Assunta Lopedota**: Formal analysis**,** Methodology, **Annalisa Cutrignelli**: Formal analysis, Data curation, **Antonio Lopalco**: Software, Validation, **Massimo Franco**: Supervision, **Antonio Pepe**: Data curation, Investigation, **Sergio Fontana**: Conceptualization, **Nunzio Denora:** Conceptualization, Writing- Reviewing and Editing.

## Declaration of Competing Interest

The authors declare that although some authors are affiliated with Farmalabor s.r.l., this did not influence their work or interpretation of results reported in this article.

## Appendix Supplementary materials

Image, application 1
