# Supplementary material for: Stability data of extemporaneous suspensions of hydroxychloroquine sulphate in oral liquid bases after tablet manipulation
Source: Data Brief. 2020 Nov 30;33:106575. doi: 10.1016/j.dib.2020.106575 (PMC8129641; doi:10.1016/j.dib.2020.106575)
Supplement: Supplementary file 1 [file mmc1.docx]

**Supplementary materials**

**Stability data of extemporaneous suspensions of hydroxychloquine sulphate in oral liquid bases after tablet manipulation**

**Authors**

Mara Perrone^a^, Valentino Laquintana^b^, Angela Assunta Lopedota^b^, Annalisa Cutrignelli^b^, Antonio Lopalco^b^, Massimo Franco^b^, Antonio Pepe^a^, Sergio Fontana^a^, Nunzio Denora^b,^*

**Affiliations**

^a^ Centro Studi e Ricerche “Dr. S. Fontana 1900-1982”, Farmalabor s.r.l., Via Piano S. Giovanni, 47, 76012 Canosa di Puglia (BT), Italy

^b^ Department of Pharmacy – Pharmaceutical Sciences, University of Bari “Aldo Moro”, Via E. Orabona, 4, 70125 Bari, Italy

**Corresponding author(s)**

* Nunzio Denora ([nunzio.denora@uniba.it](mailto:nunzio.denora@uniba.it))

**Figure S1.** Turbiscan backscattering and transmission profiles (ΔT and ΔBS) of extemporaneous suspensions of hydroxychloroquine sulphate, after tablet manipulation, in: **A)** fast oral solution “Puccini”; **B)** an enlargement of **A**; **C)** fast oral solution - sugar free “Beethoven”. Data are shown as sample height (0–40 mm) as function of time. The blue arrow indicates the direction of analysis over time.

**A)**


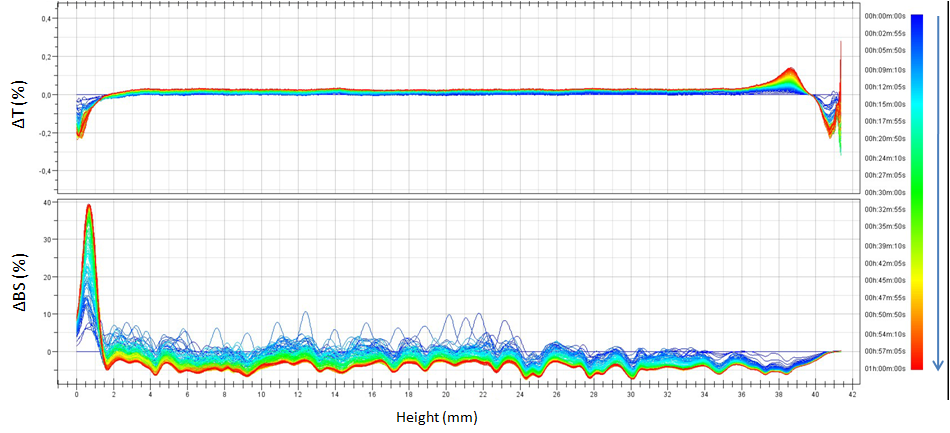


**B)**


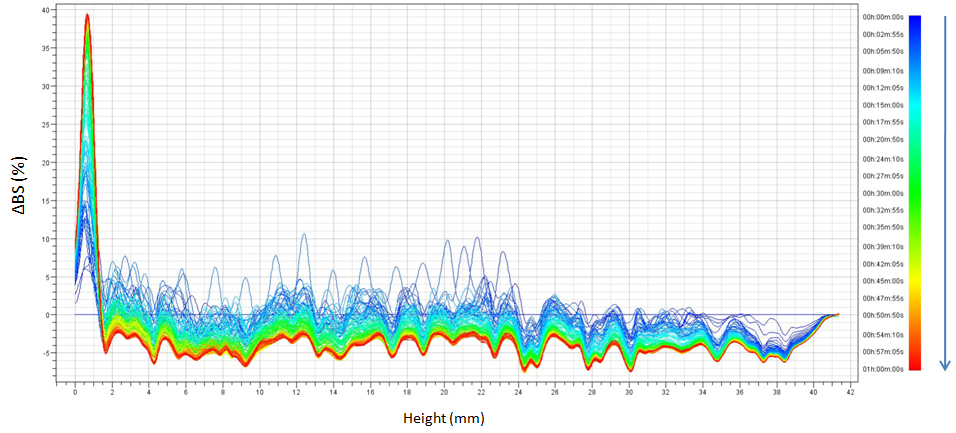


**C)**


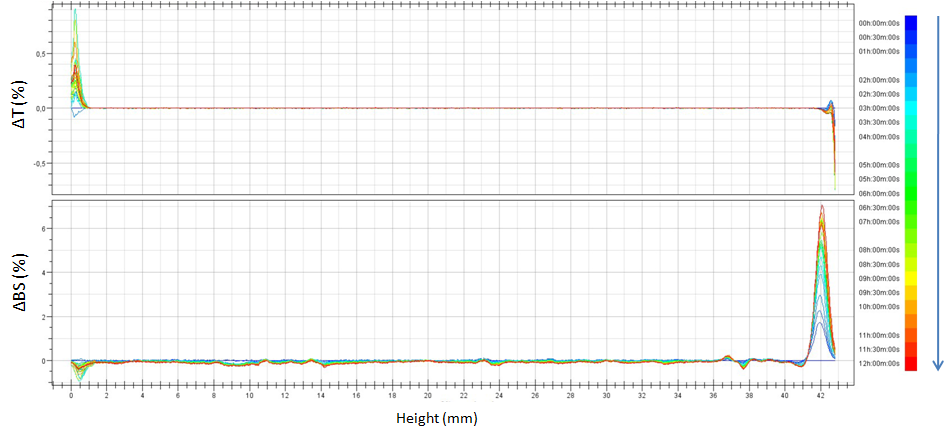


**Figure S2**. The Turbiscan Stability Index (TSI) profiles of extemporaneous suspensions of hydroxychloroquine sulphate, after tablet manipulation, in: **A)** fast oral solution “Puccini”; **B)** fast oral solution - sugar free “Beethoven”.

**A)**


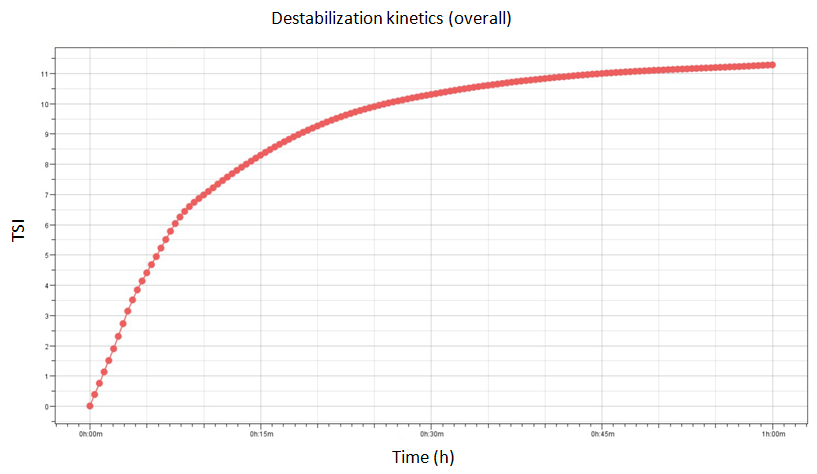


**B)**


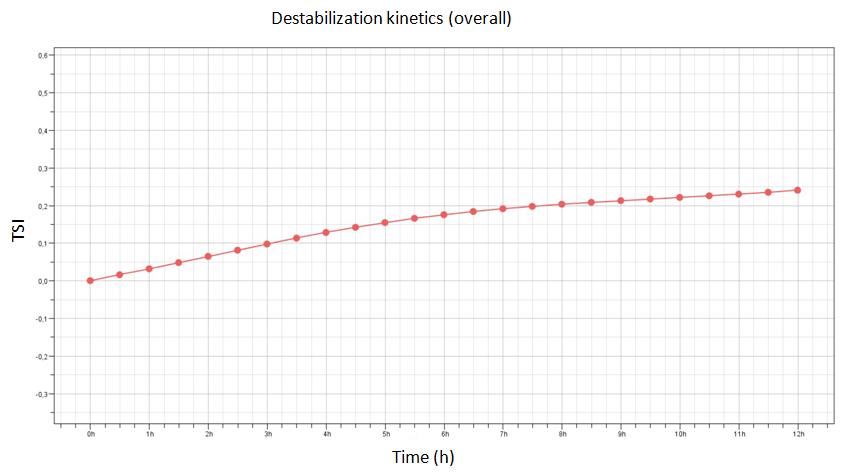


**Figure S3**. Chromatogram of control hydroxychloroquine sulphate.

**
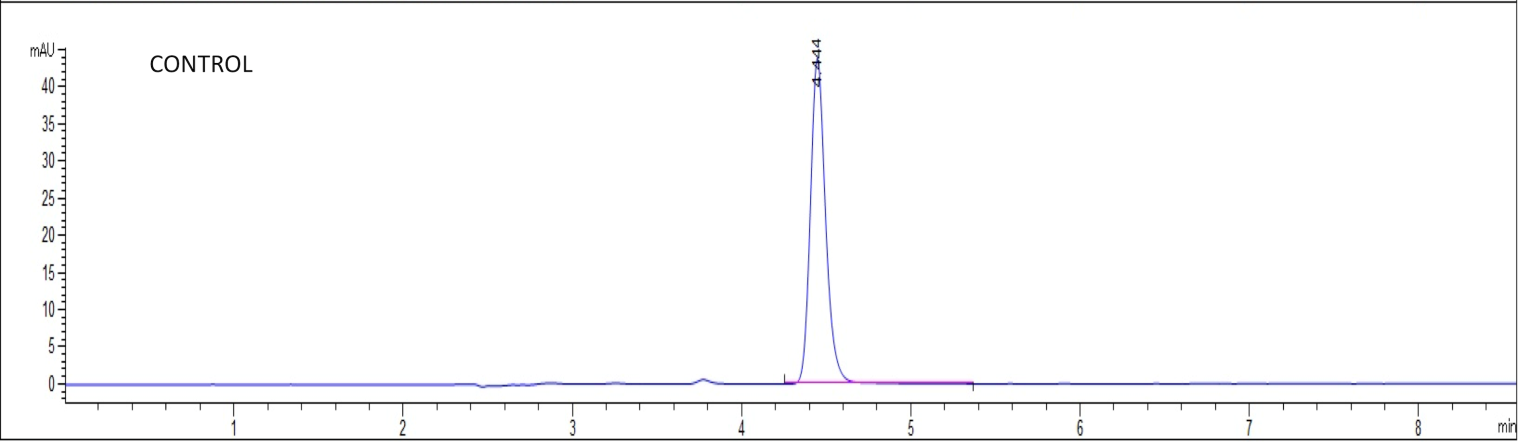
**

**Table S1**. Raw data on the chemical stability of hydroxychloroquine sulphate in fast oral solution “Puccini”-based extemporaneous suspensions when stored at RT or 4 °C for one month (30 days).

| Storage Condition (°C) | Dilution | Sampling Times (days) | Retention Time (min) | Peak Area | Concentration (mg/mL) | Mean Concentration (mg/mL) | Standard Deviation (mg/mL) |
| --- | --- | --- | --- | --- | --- | --- | --- |
| 4 | 1:200 | 0 | 4.49 | 1292.68 | 30.01 | 30.02 | 0.27 |
|  |  |  | 4.489 | 1281.48 | 29.75 |  |  |
|  |  |  | 4.473 | 1304.74 | 30.29 |  |  |
|  |  | 3 | 4.477 | 1279.33 | 29.7 | 30.05 | 0.32 |
|  |  |  | 4.492 | 1298.28 | 30.14 |  |  |
|  |  |  | 4.497 | 1306.03 | 30.32 |  |  |
|  |  | 7 | 4.499 | 1293.54 | 30.03 | 30.1 | 0.18 |
|  |  |  | 4.504 | 1290.96 | 29.97 |  |  |
|  |  |  | 4.494 | 1305.60 | 29.97 |  |  |
|  |  | 14 | 4.497 | 1282.34 | 29.77 | 29.94 | 0.17 |
|  |  |  | 4.414 | 1290.10 | 29.95 |  |  |
|  |  |  | 4.499 | 1296.99 | 30.11 |  |  |
|  |  | 21 | 4.497 | 1284.93 | 29.83 | 29.9 | 0.07 |
|  |  |  | 4.491 | 1288.37 | 29.91 |  |  |
|  |  |  | 4.507 | 1290.53 | 29.91 |  |  |
|  |  | 30 | 4.482 | 1292.25 | 30 | 29.96 | 0.07 |
|  |  |  | 4.499 | 1287.08 | 29.88 |  |  |
|  |  |  | 4.503 | 1291.82 | 29.99 |  |  |
| RT | 1:200 | 0 | 4.498 | 1287.51 | 29.89 | 30.05 | 0.18 |
|  |  |  | 4.497 | 1292.68 | 30.01 |  |  |
|  |  |  | 4.488 | 1302.59 | 30.24 |  |  |
|  |  | 3 | 4.488 | 1294.83 | 30.06 | 29.92 | 0.21 |
|  |  |  | 4.503 | 1293.54 | 30.03 |  |  |
|  |  |  | 4.509 | 1278.47 | 29.68 |  |  |
|  |  | 7 | 4.497 | 1298.28 | 30.14 | 29.91 | 0.42 |
|  |  |  | 4.496 | 1299.14 | 30.16 |  |  |
|  |  |  | 4.502 | 1267.70 | 29.43 |  |  |
|  |  | 14 | 4.5 | 1287.51 | 29.43 | 29.99 | 0.39 |
|  |  |  | 4.422 | 1278.04 | 29.67 |  |  |
|  |  |  | 4.511 | 1310.34 | 30.42 |  |  |
|  |  | 21 | 4.494 | 1297.85 | 30.13 | 30.06 | 0.06 |
|  |  |  | 4.494 | 1293.11 | 30.02 |  |  |
|  |  |  | 4.51 | 1293.97 | 30.04 |  |  |
|  |  | 30 | 4.488 | 1287.94 | 29.9 | 30.01 | 0.15 |
|  |  |  | 4.517 | 1290.10 | 29.95 |  |  |
|  |  |  | 4.502 | 1300.00 | 30.18 |  |  |

**Table S2**. Raw data on the chemical stability of hydroxychloroquine sulphate in fast oral solution - sugar free “Beethoven”-based extemporaneous suspensions when stored at RT or 4 °C for one month (30 days).

| Storage Condition (°C) | Dilution | Sampling Times (days) | Retention Time (min) | Peak Area | Concentration (mg/mL) | Mean Concentration (mg/mL) | Standard Deviation (mg/mL) |
| --- | --- | --- | --- | --- | --- | --- | --- |
| 4 | 1:200 | 0 | 4.507 | 1287.51 | 29.89 | 30.01 | 0.16 |
|  |  |  | 4.485 | 1300.43 | 30.19 |  |  |
|  |  |  | 4.497 | 1289.67 | 29.94 |  |  |
|  |  | 3 | 4.478 | 1300.43 | 30.19 | 30.12 | 0.34 |
|  |  |  | 4.507 | 1281.48 | 29.75 |  |  |
|  |  |  | 4.496 | 1309.91 | 30.41 |  |  |
|  |  | 7 | 4.498 | 1288.37 | 29.91 | 30.05 | 0.15 |
|  |  |  | 4.488 | 1293.54 | 30.03 |  |  |
|  |  |  | 4.505 | 1300.87 | 30.2 |  |  |
|  |  | 14 | 4.517 | 1281.91 | 29.76 | 29.98 | 0.22 |
|  |  |  | 4.51 | 1291.39 | 29.98 |  |  |
|  |  |  | 4.49 | 1300.43 | 30.19 |  |  |
|  |  | 21 | 4.499 | 1283.20 | 29.79 | 29.93 | 0.18 |
|  |  |  | 4.49 | 1286.65 | 29.98 |  |  |
|  |  |  | 4.498 | 1298.28 | 30.19 |  |  |
|  |  | 30 | 4.49 | 1293.11 | 30.02 | 29.96 | 0.09 |
|  |  |  | 4.498 | 1286.22 | 29.86 |  |  |
|  |  |  | 4.501 | 1291.82 | 29.99 |  |  |
| RT | 1:200 | 0 | 4.473 | 1283.20 | 29.79 | 30.04 | 0.23 |
|  |  |  | 4.497 | 1296.13 | 30.09 |  |  |
|  |  |  | 4.494 | 1302.59 | 30.24 |  |  |
|  |  | 3 | 4.493 | 1292.68 | 30.01 | 29.89 | 0.18 |
|  |  |  | 4.503 | 1291.39 | 29.98 |  |  |
|  |  |  | 4.509 | 1278.47 | 29.68 |  |  |
|  |  | 7 | 4.497 | 1293.11 | 30.02 | 29.84 | 0.34 |
|  |  |  | 4.496 | 1294.83 | 30.06 |  |  |
|  |  |  | 4.494 | 1268.56 | 29.45 |  |  |
|  |  | 14 | 4.494 | 1288.80 | 29.92 | 30.01 | 0.12 |
|  |  |  | 4.502 | 1290.53 | 29.96 |  |  |
|  |  |  | 4.502 | 1298.28 | 30.14 |  |  |
|  |  | 21 | 4.488 | 1298.71 | 30.15 | 29.99 | 0.17 |
|  |  |  | 4.491 | 1291.82 | 29.99 |  |  |
|  |  |  | 4.513 | 1284.50 | 29.82 |  |  |
|  |  | 30 | 4.504 | 1299.57 | 30.17 | 30.12 | 0.09 |
|  |  |  | 4.509 | 1292.68 | 30.01 |  |  |
|  |  |  | 4.492 | 1299.57 | 30.17 |  |  |

**Table S3.** Raw data of Turbiscan backscattering and transmission measurements (ΔT % and ΔBS (%) of extemporaneous suspensions of hydroxychloroquine sulphate, after tablet manipulation, in fast oral solution “Puccini” depicted in Fig. S1 (panel A and B) after 25 s, 30 min and 1h.

| Sample height (µm) | ΔT (%) 00h:00m:25s | ΔBS (%) 00h:00m:25s | ΔT (%) 00h:30m:00s | ΔBS (%) 00h:30m:00s | ΔT (%) 01h:00m:00s | ΔBS (%) 01h:00m:00s |
| --- | --- | --- | --- | --- | --- | --- |
| 0 | -0.033384 | 1.492123 | -0.151936 | 9.39649 | -0.201455 | 8.795444 |
| 200 | -0.02656 | 2.726015 | -0.204259 | 17.16152 | -0.198957 | 16.53707 |
| 400 | -0.055435 | 5.430351 | -0.181501 | 29.00094 | -0.147081 | 29.24068 |
| 600 | -0.032251 | 7.466936 | -0.114378 | 35.93742 | -0.104589 | 38.65935 |
| 800 | -0.020753 | 7.093801 | -0.07395 | 32.1077 | -0.074881 | 36.92832 |
| 1000 | -0.013935 | 4.91194 | -0.047139 | 20.04478 | -0.050948 | 25.31464 |
| 1200 | -0.008801 | 2.319685 | -0.028571 | 7.15785 | -0.029833 | 10.3571 |
| 1400 | -0.007902 | -0.71255 | -0.015265 | -1.20845 | -0.013315 | -0.50079 |
| 1600 | -0.007215 | -1.9235 | -0.007638 | -4.16732 | -0.003596 | -4.80465 |
| 1800 | -0.007724 | -0.71703 | -0.005288 | -3.18397 | 0.002197 | -4.41608 |
| 2000 | -0.009204 | 0.758542 | -0.00348 | -1.59875 | 0.004197 | -3.1397 |
| 2200 | -0.006834 | 1.676646 | 0.001878 | -0.96695 | 0.009908 | -2.27272 |
| 2400 | -0.005491 | 1.524731 | 0.0037 | -1.11911 | 0.01159 | -2.05476 |
| 2600 | -0.007052 | 0.504256 | 0.003931 | -1.60125 | 0.012614 | -2.49422 |
| 2800 | -0.001202 | -0.09055 | 0.009891 | -1.9495 | 0.019627 | -3.00812 |
| 3000 | 0.003038 | 0.956999 | 0.017547 | -1.50929 | 0.026868 | -2.87554 |
| 3200 | -0.001242 | 3.053051 | 0.011221 | -1.04017 | 0.023799 | -2.65996 |
| 3400 | 0.000098 | 3.252306 | 0.01555 | -1.13628 | 0.025997 | -2.93423 |
| 3600 | 0.002759 | 1.01223 | 0.016738 | -1.61068 | 0.028669 | -3.79308 |
| 3800 | 0.004931 | -0.01905 | 0.018532 | -1.93674 | 0.027514 | -4.39314 |
| 4000 | 0.00116 | -0.88204 | 0.018427 | -2.96171 | 0.027751 | -5.10543 |
| 4200 | 0.001389 | -2.28749 | 0.020293 | -4.61386 | 0.028676 | -6.24491 |
| 4400 | -0.000781 | -1.27237 | 0.02014 | -4.11727 | 0.02756 | -5.75854 |
| 4600 | -0.002915 | 1.376006 | 0.016883 | -1.95884 | 0.02652 | -3.91715 |
| 4800 | -0.003586 | 2.612522 | 0.018056 | -0.90886 | 0.024615 | -3.03133 |
| 5000 | -0.002824 | 2.392509 | 0.016731 | -1.51072 | 0.025513 | -3.12063 |
| 5200 | 0.000413 | 1.7537 | 0.0186 | -2.72437 | 0.026268 | -3.82713 |
| 5400 | 0.002783 | 0.756763 | 0.022296 | -3.67785 | 0.030631 | -4.82723 |
| 5600 | 0.000859 | -0.68173 | 0.019152 | -4.41001 | 0.027025 | -5.5697 |
| 5800 | -0.000312 | -1.60748 | 0.018543 | -4.31556 | 0.026583 | -5.33583 |
| 6000 | -0.000292 | -2.0112 | 0.019665 | -4.10274 | 0.026696 | -5.09023 |
| 6400 | 0.002467 | -2.18589 | 0.020718 | -4.28166 | 0.029392 | -5.7156 |
| 6600 | 0.001974 | -1.3667 | 0.021838 | -4.69694 | 0.030489 | -5.80623 |
| 6800 | 0.001925 | 0.376154 | 0.023557 | -4.66334 | 0.031486 | -5.36882 |
| 7000 | 0.000172 | 1.478377 | 0.022115 | -4.37938 | 0.029591 | -4.99534 |
| 7200 | 0.000948 | 1.237283 | 0.02434 | -4.14153 | 0.031031 | -4.72483 |
| 7400 | 0.001171 | 1.038499 | 0.02236 | -4.07508 | 0.028807 | -4.5723 |
| 7600 | 0.004162 | 1.183086 | 0.023758 | -4.25076 | 0.030285 | -4.90604 |
| 7800 | 0.000852 | 0.808556 | 0.023139 | -4.40039 | 0.026762 | -5.29653 |
| 8000 | -0.00048 | 0.811611 | 0.02355 | -4.03346 | 0.027657 | -4.81761 |
| 8200 | 0.000011 | 0.936744 | 0.025336 | -3.99723 | 0.02879 | -4.32041 |
| 8400 | -0.002464 | 0.255412 | 0.019688 | -4.50024 | 0.024179 | -4.99618 |
| 8600 | -0.000346 | -0.22617 | 0.02138 | -4.43629 | 0.026319 | -5.34124 |
| 8800 | -0.000395 | -0.8307 | 0.021066 | -4.48159 | 0.025452 | -5.64619 |
| 9000 | 0.001612 | -2.08693 | 0.022344 | -5.14166 | 0.027846 | -6.34301 |
| 9200 | 0.002128 | -3.22515 | 0.020424 | -5.59358 | 0.026065 | -6.74715 |
| 9400 | 0.001978 | -3.04685 | 0.019137 | -5.10007 | 0.027001 | -6.23309 |
| 9600 | 0.003091 | -2.29992 | 0.02071 | -4.26354 | 0.027703 | -5.61242 |
| 9800 | 0.000286 | -1.5014 | 0.021138 | -3.56528 | 0.02825 | -4.77086 |
| 10000 | 0.000471 | -1.24705 | 0.017397 | -2.87424 | 0.022316 | -3.81126 |
| 10200 | 0.001311 | -1.14871 | 0.017969 | -2.48939 | 0.023824 | -3.15959 |
| 10400 | 0.000386 | -0.59812 | 0.017003 | -2.38796 | 0.024971 | -3.16081 |
| 10600 | 0.000723 | 0.011866 | 0.021279 | -2.20249 | 0.026866 | -3.16275 |
| 10800 | -0.001331 | 0.193991 | 0.019062 | -1.94312 | 0.027565 | -3.30671 |
| 11000 | -0.000609 | 0.357169 | 0.020412 | -1.51563 | 0.029765 | -3.10748 |
| 11040 | -0.00021 | 0.407586 | 0.017929 | -1.47883 | 0.025138 | -3.08206 |
| 11080 | -0.001974 | 0.490347 | 0.018012 | -1.45056 | 0.025414 | -3.02442 |
| 11120 | -0.002473 | 0.530429 | 0.019115 | -1.46049 | 0.027783 | -2.97313 |
| 11160 | -0.003294 | 0.543313 | 0.018821 | -1.51911 | 0.027134 | -2.94141 |
| 11200 | -0.0036 | 0.599895 | 0.018489 | -1.53335 | 0.026481 | -2.89936 |
| 11240 | -0.003997 | 0.616397 | 0.017181 | -1.58037 | 0.025641 | -2.88007 |
| 11280 | -0.002685 | 0.638296 | 0.01826 | -1.61403 | 0.029349 | -2.82387 |
| 11320 | -0.002625 | 0.632185 | 0.017573 | -1.68912 | 0.027059 | -2.85122 |
| 11360 | -0.002621 | 0.587216 | 0.018517 | -1.77389 | 0.026544 | -2.83818 |
| 11400 | -0.002578 | 0.527832 | 0.020366 | -1.85546 | 0.025832 | -2.87183 |
| 11440 | -0.002259 | 0.465597 | 0.020369 | -1.94407 | 0.026338 | -2.90041 |
| 11480 | -0.001187 | 0.357169 | 0.019738 | -2.03889 | 0.026457 | -2.96208 |
| 11520 | -0.002429 | 0.215789 | 0.017694 | -2.15068 | 0.025576 | -3.0542 |
| 11560 | -0.003778 | 0.108735 | 0.01705 | -2.20424 | 0.024387 | -3.08742 |
| 11600 | -0.001597 | -0.01324 | 0.019286 | -2.22785 | 0.026125 | -3.15705 |
| 11800 | -0.002128 | -0.22205 | 0.017855 | -1.88381 | 0.021212 | -3.26819 |
| 12000 | 0.000582 | 0.154163 | 0.018726 | -0.99639 | 0.024978 | -2.88024 |
| 12200 | -0.002112 | 0.935822 | 0.017854 | -0.69159 | 0.026672 | -2.47311 |
| 12240 | -0.002754 | 1.083314 | 0.01679 | -0.7461 | 0.022906 | -2.43387 |
| 12280 | -0.001706 | 1.236204 | 0.016889 | -0.80183 | 0.025026 | -2.39635 |
| 12320 | -0.000598 | 1.33241 | 0.018251 | -0.91085 | 0.0237 | -2.39115 |
| 12360 | -0.001296 | 1.379013 | 0.018303 | -1.02544 | 0.021989 | -2.41011 |
| 12400 | -0.001633 | 1.392813 | 0.018885 | -1.15116 | 0.024559 | -2.451 |
| 12600 | -0.000227 | 1.196278 | 0.018137 | -1.72454 | 0.022539 | -2.8898 |
| 12800 | 0.001691 | 0.519678 | 0.022081 | -2.71549 | 0.026684 | -3.80789 |
| 13000 | 0.000406 | 0.154001 | 0.022241 | -4.04513 | 0.026936 | -5.00867 |
| 13200 | -0.001015 | 0.64313 | 0.024055 | -4.89198 | 0.027287 | -5.46028 |
| 13400 | 0.001077 | 1.015485 | 0.025371 | -4.45986 | 0.028891 | -4.70211 |
| 13600 | 0.000593 | 0.482561 | 0.025076 | -4.12615 | 0.027575 | -4.26128 |
| 13800 | 0.003669 | -0.21721 | 0.027464 | -4.27555 | 0.032042 | -4.62044 |
| 14000 | 0.004139 | -0.70186 | 0.02835 | -4.64165 | 0.031108 | -5.1991 |
| 14200 | 0.005879 | -1.01869 | 0.027574 | -5.09754 | 0.030544 | -5.66569 |
| 14400 | 0.005002 | -0.95177 | 0.026074 | -5.13876 | 0.030392 | -5.76594 |
| 14600 | 0.003258 | -0.77952 | 0.02299 | -4.62593 | 0.029724 | -5.40518 |
| 14800 | 0.000198 | -0.52498 | 0.021384 | -3.85076 | 0.026309 | -4.57236 |
| 15000 | 0.001612 | -0.44899 | 0.022412 | -3.15465 | 0.026628 | -3.85896 |
| 15200 | 0.000694 | -0.58161 | 0.020015 | -2.8236 | 0.025552 | -3.78816 |
| 15400 | 0.000042 | -0.60168 | 0.020198 | -2.85552 | 0.026292 | -3.92682 |
| 15600 | -0.000841 | -0.51067 | 0.020123 | -3.08298 | 0.025963 | -4.01259 |
| 15800 | -0.001539 | -0.14928 | 0.02052 | -3.00637 | 0.024124 | -3.74318 |
| 16000 | -0.00401 | 0.233814 | 0.018261 | -2.81183 | 0.020767 | -3.21397 |
| 16200 | -0.00281 | 0.45144 | 0.017633 | -2.63616 | 0.019678 | -2.56512 |
| 16400 | -0.003308 | 0.112965 | 0.019631 | -2.54953 | 0.019992 | -2.2216 |
| 16600 | -0.002556 | -0.54759 | 0.016831 | -2.5423 | 0.020353 | -2.53141 |
| 16800 | 0.000261 | -0.756 | 0.020605 | -3.04132 | 0.022737 | -3.57259 |
| 17000 | -0.000016 | -0.42655 | 0.01779 | -4.32821 | 0.025215 | -5.04614 |
| 17200 | -0.000288 | -0.22775 | 0.018258 | -4.86492 | 0.021612 | -5.50234 |
| 17400 | 0.005188 | -0.15141 | 0.021888 | -4.12759 | 0.027345 | -4.84956 |
| 17600 | 0.002886 | 0.798625 | 0.019004 | -2.77672 | 0.023739 | -3.75423 |
| 17800 | 0.002875 | 1.152436 | 0.020477 | -1.97935 | 0.026827 | -3.22646 |
| 18000 | 0.002121 | 0.527838 | 0.018479 | -1.80391 | 0.023574 | -3.22309 |
| 18200 | 0.004302 | 0.234428 | 0.021386 | -1.90001 | 0.024017 | -3.29842 |
| 18400 | 0.003655 | 0.089482 | 0.01945 | -2.2257 | 0.025155 | -3.52196 |
| 18600 | 0.004224 | -0.62124 | 0.020597 | -3.27788 | 0.025312 | -4.42839 |
| 18800 | 0.003767 | -2.09329 | 0.021044 | -4.27015 | 0.025822 | -5.28808 |
| 19000 | -0.000198 | -2.47598 | 0.018257 | -3.99331 | 0.022643 | -4.72604 |
| 19200 | 0.000631 | -1.35039 | 0.018363 | -2.80775 | 0.022471 | -3.26299 |
| 19400 | -0.001956 | -0.21248 | 0.021432 | -2.04911 | 0.022071 | -2.43907 |
| 19600 | -0.004708 | 0.616288 | 0.018369 | -2.00256 | 0.020298 | -2.34501 |
| 19800 | -0.005915 | 1.252909 | 0.017571 | -1.86267 | 0.023143 | -2.45813 |
| 20000 | -0.006142 | 1.012375 | 0.019613 | -1.96127 | 0.022754 | -2.67764 |
| 20200 | -0.003339 | 0.213956 | 0.022526 | -2.37783 | 0.024155 | -3.01453 |
| 20400 | -0.003334 | -0.46855 | 0.0245 | -2.92355 | 0.025348 | -3.54827 |
| 20600 | -0.003644 | -0.04625 | 0.02159 | -2.96977 | 0.024279 | -3.51024 |
| 20800 | -0.004503 | 0.680976 | 0.020703 | -2.70096 | 0.023722 | -3.04813 |
| 21000 | -0.00188 | 0.823272 | 0.024285 | -2.70106 | 0.025155 | -2.88156 |
| 21200 | -0.001617 | 0.855103 | 0.026331 | -2.73516 | 0.026938 | -2.73368 |
| 21400 | -0.002882 | 0.782684 | 0.022584 | -2.52477 | 0.025576 | -2.44614 |
| 21600 | -0.002986 | 0.597504 | 0.022177 | -2.38191 | 0.024455 | -2.27416 |
| 21800 | -0.00172 | 0.358031 | 0.023033 | -2.64918 | 0.024233 | -2.52986 |
| 22000 | -0.00236 | 0.330888 | 0.022804 | -2.88028 | 0.026691 | -2.82232 |
| 22200 | -0.001628 | 0.352179 | 0.021892 | -2.78444 | 0.023318 | -2.86697 |
| 22400 | -0.001068 | -0.42398 | 0.022864 | -3.03166 | 0.027054 | -3.53981 |
| 22600 | 0.000803 | -1.65974 | 0.023206 | -4.09859 | 0.027064 | -4.99618 |
| 22800 | 0.003073 | -2.02205 | 0.022467 | -4.37931 | 0.0269 | -5.40165 |
| 23000 | 0.004387 | -0.86661 | 0.025609 | -2.97938 | 0.029387 | -4.27559 |
| 23200 | 0.001574 | 0.229228 | 0.022398 | -2.1078 | 0.028022 | -3.24055 |
| 23400 | 0.001345 | 1.437976 | 0.021903 | -1.89081 | 0.027318 | -2.75169 |
| 23600 | -0.000286 | 2.981691 | 0.021956 | -1.97152 | 0.025058 | -2.79961 |
| 23800 | -0.003165 | 4.046731 | 0.020653 | -2.43285 | 0.023848 | -3.41558 |
| 24000 | -0.000941 | 3.328029 | 0.023149 | -3.72686 | 0.026246 | -4.74599 |
| 24200 | 0.000317 | 0.829804 | 0.02552 | -5.70621 | 0.028361 | -6.4836 |
| 24400 | 0.000194 | -1.01807 | 0.025076 | -6.1338 | 0.027655 | -6.82336 |
| 24600 | -0.000484 | -1.02658 | 0.023499 | -5.28695 | 0.026771 | -5.93312 |
| 24800 | -0.002946 | -1.40947 | 0.022516 | -5.27932 | 0.026389 | -6.01331 |
| 25000 | 0.000453 | -2.60849 | 0.024398 | -5.58059 | 0.028826 | -6.57191 |
| 25200 | 0.001099 | -2.34117 | 0.023647 | -4.98545 | 0.026912 | -5.93035 |
| 25400 | -0.000129 | -0.74286 | 0.021604 | -3.53226 | 0.025307 | -4.43829 |
| 25600 | -0.00031 | 0.364961 | 0.022169 | -2.448 | 0.026769 | -3.37844 |
| 25800 | -0.002224 | 0.359007 | 0.021114 | -1.93091 | 0.022858 | -2.81724 |
| 26000 | 0.000016 | 0.01849 | 0.021272 | -2.11769 | 0.023219 | -2.82547 |
| 26200 | 0.001204 | -0.42134 | 0.02195 | -2.59591 | 0.024956 | -3.08521 |
| 26400 | 0.001992 | -0.76847 | 0.02217 | -3.00269 | 0.024259 | -3.41795 |
| 26600 | 0.000787 | -1.08852 | 0.022625 | -3.39749 | 0.02576 | -3.78192 |
| 26800 | 0.004579 | -1.2825 | 0.023887 | -3.78464 | 0.028848 | -4.25442 |
| 27000 | 0.001456 | -0.70238 | 0.02337 | -3.5082 | 0.026907 | -4.19081 |
| 27200 | 0.002572 | 0.721197 | 0.024804 | -3.06897 | 0.027698 | -3.90217 |
| 27400 | 0.001889 | 1.805077 | 0.024746 | -3.54163 | 0.028298 | -4.59568 |
| 27600 | 0.002444 | 0.64146 | 0.026651 | -5.44502 | 0.028214 | -6.47697 |
| 27800 | 0.007541 | -1.43207 | 0.030312 | -6.55007 | 0.033168 | -7.24961 |
| 28000 | 0.004084 | -1.41268 | 0.029215 | -5.84529 | 0.03264 | -6.32648 |
| 28200 | 0.006095 | -0.74607 | 0.025807 | -5.4326 | 0.029976 | -5.91361 |
| 28400 | 0.002259 | -0.94912 | 0.025426 | -5.63527 | 0.02742 | -6.24116 |
| 28600 | 0.002092 | -0.8302 | 0.024802 | -5.16609 | 0.027628 | -5.76014 |
| 28800 | 0.003459 | -0.23066 | 0.025023 | -4.22333 | 0.027691 | -4.58225 |
| 29000 | 0.000812 | 0.189405 | 0.021963 | -3.61284 | 0.024158 | -3.64884 |
| 29200 | 0.000143 | 0.794339 | 0.022693 | -3.36859 | 0.024956 | -3.38297 |
| 29400 | -0.000446 | 1.058563 | 0.023943 | -3.66368 | 0.025525 | -3.7171 |
| 29600 | 0.001702 | 0.253527 | 0.02452 | -4.60518 | 0.027681 | -4.78451 |
| 29800 | 0.003381 | -1.21756 | 0.028507 | -5.72226 | 0.029755 | -6.24475 |
| 29840 | 0.002721 | -1.52981 | 0.026963 | -5.94028 | 0.029888 | -6.52085 |
| 29880 | 0.003082 | -1.82332 | 0.025873 | -6.1533 | 0.027577 | -6.78462 |
| 29920 | 0.003832 | -2.03427 | 0.026054 | -6.29086 | 0.028395 | -6.95931 |
| 29960 | 0.001869 | -2.27562 | 0.025074 | -6.45969 | 0.028487 | -7.14593 |
| 30000 | 0.00174 | -2.4936 | 0.026612 | -6.57276 | 0.029358 | -7.28139 |
| 30200 | 0.003903 | -2.89665 | 0.026723 | -6.32071 | 0.030041 | -7.08724 |
| 30400 | 0.003716 | -1.96846 | 0.025306 | -4.62859 | 0.029717 | -5.41911 |
| 30600 | 0.001789 | -1.70527 | 0.023413 | -4.06486 | 0.027584 | -4.65321 |
| 30800 | 0.001978 | -1.87415 | 0.024344 | -4.39196 | 0.027035 | -4.96269 |
| 31000 | 0.001095 | -1.23285 | 0.024451 | -4.34814 | 0.027521 | -4.91881 |
| 31200 | -0.000301 | -0.52773 | 0.021003 | -4.25187 | 0.02523 | -4.82104 |
| 31400 | 0.001517 | -0.24746 | 0.025702 | -4.18947 | 0.029404 | -4.65453 |
| 31600 | 0.002424 | -0.25898 | 0.026611 | -4.02846 | 0.027505 | -4.36749 |
| 31800 | -0.000874 | -0.04131 | 0.024341 | -3.83113 | 0.025839 | -4.15932 |
| 32000 | -0.005201 | 0.290855 | 0.019601 | -3.81051 | 0.022989 | -4.12815 |
| 32200 | -0.003506 | 0.675727 | 0.022309 | -3.88034 | 0.021863 | -4.2053 |
| 32400 | -0.003149 | 0.808351 | 0.02166 | -4.11289 | 0.024283 | -4.36572 |
| 32600 | -0.000357 | 0.41304 | 0.025718 | -4.34028 | 0.028114 | -4.54478 |
| 32800 | -0.000223 | -0.19852 | 0.024027 | -4.4882 | 0.026754 | -4.67117 |
| 33000 | 0.000359 | -0.76332 | 0.024749 | -4.3845 | 0.026355 | -4.54323 |
| 33200 | 0.00145 | -1.09253 | 0.025087 | -4.18049 | 0.026357 | -4.43127 |
| 33400 | 0.003986 | -0.88816 | 0.026625 | -3.90007 | 0.027297 | -4.17739 |
| 33600 | 0.00132 | -0.48444 | 0.023649 | -3.59125 | 0.027253 | -3.9014 |
| 33800 | 0.002413 | -0.22022 | 0.024114 | -3.31316 | 0.02734 | -3.7061 |
| 34000 | 0.00227 | -0.14474 | 0.025986 | -3.37766 | 0.028942 | -3.77866 |
| 34200 | 0.001073 | -0.19787 | 0.025189 | -3.95057 | 0.029281 | -4.30007 |
| 34400 | -0.001713 | -0.27905 | 0.02282 | -4.69737 | 0.028327 | -5.09907 |
| 34600 | -0.00058 | -0.48454 | 0.02762 | -5.47514 | 0.031142 | -5.88415 |
| 34800 | -0.002166 | -0.6084 | 0.02713 | -5.72726 | 0.030665 | -6.08299 |
| 35000 | 0.001104 | -0.60478 | 0.030079 | -5.2039 | 0.033782 | -5.5114 |
| 35200 | -0.001682 | -0.55686 | 0.025647 | -4.41166 | 0.029129 | -4.76479 |
| 35400 | -0.003027 | -0.23473 | 0.02411 | -3.60515 | 0.028795 | -3.99949 |
| 35600 | -0.001519 | -0.08643 | 0.023891 | -3.19476 | 0.027839 | -3.61834 |
| 35800 | -0.001686 | -0.23422 | 0.025189 | -3.16251 | 0.029755 | -3.51715 |
| 36000 | -0.000292 | -0.30517 | 0.025529 | -3.33377 | 0.032352 | -3.7371 |
| 36200 | 0.000709 | -0.15076 | 0.029402 | -3.71664 | 0.035835 | -4.16479 |
| 36400 | 0.001599 | -0.02746 | 0.031843 | -4.24276 | 0.038969 | -4.67294 |
| 36600 | 0.002786 | -0.17499 | 0.036065 | -4.46621 | 0.043214 | -4.91223 |
| 36800 | 0.009744 | -0.57382 | 0.042702 | -4.56609 | 0.053458 | -5.00231 |
| 37000 | 0.004333 | -0.90247 | 0.039059 | -5.08057 | 0.051713 | -5.52765 |
| 37200 | 0.007621 | -1.27395 | 0.043782 | -5.75708 | 0.058976 | -6.2017 |
| 37400 | 0.007429 | -1.16272 | 0.046062 | -5.62744 | 0.066222 | -6.05182 |
| 37600 | 0.006345 | -0.52498 | 0.047448 | -5.11174 | 0.071374 | -5.51521 |
| 37800 | 0.004951 | -0.5371 | 0.049648 | -5.14541 | 0.081372 | -5.513 |
| 38000 | 0.006626 | -0.88469 | 0.057496 | -5.17661 | 0.094114 | -5.56351 |
| 38200 | 0.006363 | -1.78471 | 0.058072 | -5.44684 | 0.111729 | -5.82977 |
| 38400 | 0.008243 | -2.60896 | 0.066947 | -5.83766 | 0.129355 | -6.21833 |
| 38600 | 0.006249 | -2.26539 | 0.065021 | -5.45443 | 0.138094 | -5.80452 |
| 38800 | 0.007768 | -1.62515 | 0.062923 | -4.51802 | 0.134367 | -4.82066 |
| 39000 | 0.004706 | -1.72711 | 0.049503 | -4.05167 | 0.102879 | -4.30764 |
| 39200 | 0.003553 | -1.65673 | 0.034261 | -3.77665 | 0.06595 | -3.97037 |
| 39400 | 0.001013 | -1.59149 | 0.016401 | -3.55692 | 0.030837 | -3.71914 |
| 39600 | 0.00029 | -1.42694 | 0.002397 | -3.18559 | 0.007277 | -3.40088 |
| 39800 | -0.00116 | -1.16552 | -0.005412 | -2.71789 | -0.006941 | -3.00464 |
| 39840 | 0.000511 | -1.10577 | -0.00602 | -2.61653 | -0.005789 | -2.89096 |
| 39880 | 0.000212 | -1.02128 | -0.006583 | -2.50099 | -0.009487 | -2.78634 |
| 39920 | -0.00294 | -1.00473 | -0.01131 | -2.4451 | -0.016387 | -2.70096 |
| 39960 | -0.002694 | -0.9261 | -0.015765 | -2.30622 | -0.019504 | -2.57916 |
| 40000 | -0.003977 | -0.89111 | -0.02095 | -2.2282 | -0.025157 | -2.50268 |
| 40200 | -0.00687 | -0.65928 | -0.050833 | -1.65664 | -0.060311 | -1.92224 |
| 40400 | -0.017223 | -0.31148 | -0.097494 | -1.00702 | -0.110252 | -1.23913 |
| 40600 | -0.012964 | -0.11632 | -0.134642 | -0.48751 | -0.15059 | -0.68356 |
| 40800 | -0.004864 | -0.04334 | -0.159652 | -0.30333 | -0.177431 | -0.38541 |
| 41000 | 0.01578 | 0.050676 | -0.137458 | -0.18543 | -0.128445 | -0.14507 |
| 41200 | 0.000272 | 0.034989 | -0.078219 | -0.02128 | -0.055429 | 0.011773 |
| 41360 | -0.018655 | 0.028162 | -0.051074 | 0.186613 | 0.238205 | 0.167289 |

**Table S4.** Raw data of Turbiscan backscattering and transmission measurements (ΔT % and ΔBS (%) of extemporaneous suspensions of hydroxychloroquine sulphate, after tablet manipulation, in fast oral solution - sugar free “Beethoven” depicted in Fig. S1 (panel C), after 30 min, 6 h and 12 h.

| Sample height (µm) | ΔT (%) 00h:30m:00s | ΔBS (%) 00h:30m:00s | ΔT (%) 06h:00m:00s | ΔBS (%) 06h:00m:00s | ΔT (%) 12h:00m:00s | ΔBS (%) 12h:00m:00s |
| --- | --- | --- | --- | --- | --- | --- |
| 0 | 0.101466 | -0.08942 | 0.258675 | -0.0961 | 0.23312 | -0.08939 |
| 200 | 0.203411 | -0.14565 | 0.408673 | -0.2118 | 0.377378 | -0.20236 |
| 400 | 0.09436 | -0.28543 | 0.231829 | -0.37919 | 0.215907 | -0.39302 |
| 600 | 0.034834 | -0.20472 | 0.081223 | -0.36353 | 0.047256 | -0.29347 |
| 800 | 0.004967 | -0.08238 | 0.023492 | -0.25127 | 0.017084 | -0.2149 |
| 1000 | 0.001066 | -0.03668 | 0.002428 | -0.16349 | 0.003579 | -0.16139 |
| 1200 | 0.001964 | -0.01356 | 0.001544 | -0.15779 | 0.001544 | -0.11131 |
| 1400 | -0.00042 | -0.00396 | 0.001793 | -0.09907 | -6.6E-05 | -0.0964 |
| 1600 | -7.9E-05 | -0.01783 | 0.00094 | -0.10367 | -0.00014 | -0.11214 |
| 1800 | -0.00135 | -0.03053 | -0.00168 | -0.10164 | -0.00182 | -0.10743 |
| 2000 | 0.000966 | -0.01573 | 0.000645 | -0.09037 | 0.000047 | -0.12203 |
| 2200 | 0.000779 | -0.00274 | 0.000181 | -0.10565 | 0.00182 | -0.11421 |
| 2400 | 0.00079 | -0.03235 | -0.00013 | -0.13221 | -0.00014 | -0.16465 |
| 2600 | -0.00149 | -0.02808 | 0.000116 | -0.11635 | -0.00107 | -0.14954 |
| 3000 | 0.000858 | -0.02573 | 0.000152 | -0.10073 | 0.000523 | -0.12044 |
| 3200 | -0.00226 | -0.03068 | -0.00277 | -0.1022 | -0.0035 | -0.11005 |
| 3400 | -0.00074 | -0.01794 | 0.000809 | -0.05426 | -0.00045 | -0.06123 |
| 3600 | -0.00096 | -0.00186 | -0.00037 | -0.04941 | -0.00073 | -0.06957 |
| 3800 | 0.00071 | 0.029803 | 0.000342 | -0.04337 | 0.000022 | -0.07779 |
| 4000 | -0.00075 | -0.00019 | -0.00273 | -0.0511 | -0.00066 | -0.07994 |
| 4200 | -0.00091 | -0.00804 | -0.00102 | -0.05827 | 0.000156 | -0.08055 |
| 4400 | -0.00176 | -0.01156 | -0.00086 | -0.05977 | -0.00287 | -0.09613 |
| 4600 | 0.001084 | 0.028916 | 0.000138 | -0.04673 | -0.00032 | -0.08471 |
| 4800 | -0.00144 | -0.00857 | -0.00138 | -0.08111 | -0.00139 | -0.11272 |
| 5000 | 0.002575 | 0.026904 | 0.000694 | -0.04243 | 0.000592 | -0.07898 |
| 5200 | 0.002867 | 0.008182 | 0.004058 | -0.05082 | 0.003779 | -0.08887 |
| 5400 | -0.00041 | 0.000928 | 0.001639 | -0.05514 | 0.00016 | -0.09245 |
| 5600 | -0.00059 | 0.000492 | -0.00052 | -0.08806 | -0.00084 | -0.12722 |
| 5800 | -0.00119 | 0.039205 | 0.000381 | -0.03955 | 0.000637 | -0.07429 |
| 6000 | 0.000985 | -0.02269 | 0.000354 | -0.06036 | 0.001342 | -0.10387 |
| 6200 | 0.001552 | 0.000928 | 0.00105 | -0.05167 | 0.003101 | -0.09086 |
| 6400 | 0.001758 | 0.043083 | 0.001073 | -0.05311 | 0.001775 | -0.08982 |
| 6600 | 0.002052 | 0.013625 | 0.002123 | -0.07103 | 0.001888 | -0.13663 |
| 6800 | -0.00112 | -0.01221 | -0.00046 | -0.10252 | 0.000021 | -0.15373 |
| 7000 | 0.000695 | 0.005094 | 0.00173 | -0.1019 | 0.00073 | -0.13504 |
| 7200 | -6.9E-05 | -0.00142 | 0.000051 | -0.10652 | 0.000541 | -0.15118 |
| 7400 | 0.001075 | 0.013772 | 0.001821 | -0.09294 | 0.001165 | -0.13403 |
| 7600 | 0.000058 | -0.00779 | -0.00012 | -0.10652 | -0.00041 | -0.15166 |
| 7800 | 0.001195 | 0.00142 | 0.001354 | -0.08061 | -0.00084 | -0.15063 |
| 8000 | -0.00041 | 0.016123 | -0.00033 | -0.07329 | 0.000342 | -0.09817 |
| 8200 | -0.00036 | -0.00971 | 0.000995 | -0.05853 | 0.001163 | -0.07853 |
| 8400 | 0.001489 | -0.01363 | -0.00034 | -0.09032 | 0.001848 | -0.14235 |
| 8600 | 0.000118 | -0.00118 | -0.00185 | -0.19624 | -0.00432 | -0.23684 |
| 8800 | -0.0015 | -0.00612 | -0.00054 | -0.21621 | -0.00066 | -0.27061 |
| 9000 | -0.0005 | 0.009953 | -0.00011 | -0.18933 | -0.00057 | -0.25966 |
| 9200 | -0.0003 | -0.03543 | -0.00102 | -0.21731 | 0.000638 | -0.24986 |
| 9400 | 0.000431 | -0.04333 | -0.00013 | -0.22191 | 0.00043 | -0.26499 |
| 9600 | -0.00078 | -0.03696 | -0.00064 | -0.19133 | -0.0003 | -0.24317 |
| 9800 | -0.00093 | 0.006515 | -0.00073 | -0.15522 | 0.000387 | -0.20734 |
| 10000 | -3.4E-05 | 0.00975 | 0.00033 | -0.12149 | 0.000617 | -0.16255 |
| 10200 | -0.00014 | -0.02093 | -0.00249 | -0.155 | -0.00136 | -0.18852 |
| 10400 | -0.00104 | -0.03033 | -0.00109 | -0.16316 | -0.00127 | -0.2149 |
| 10600 | 0.000374 | 0.022202 | 0.000365 | -0.04449 | -0.00059 | -0.10538 |
| 10800 | 0.000112 | 0.044057 | 0.000098 | 0.036362 | -0.00048 | -0.03072 |
| 11000 | -0.00341 | 0.021464 | -0.00265 | -0.00056 | -0.00298 | -0.02708 |
| 11200 | 0.000011 | -0.02921 | 0.001238 | -0.06897 | 0.000298 | -0.12157 |
| 11400 | -0.00226 | -0.03911 | -0.00033 | -0.13625 | 0.000885 | -0.16933 |
| 11600 | -0.00051 | -0.0125 | 0.000215 | -0.11087 | 0.001501 | -0.15433 |
| 11800 | -0.00114 | 0.012201 | -0.0008 | -0.08922 | 0.000906 | -0.14233 |
| 12000 | -0.00239 | -0.01505 | -0.0019 | -0.10996 | -0.00132 | -0.1683 |
| 12200 | 0.001159 | 0.018719 | 0.000381 | -0.06434 | 0.00025 | -0.10233 |
| 12400 | -0.00267 | 0.004803 | -0.00216 | -0.07358 | -0.00301 | -0.09923 |
| 12600 | -0.00023 | 0.017151 | -0.00013 | -0.08659 | -0.00142 | -0.11121 |
| 12800 | 0.001972 | -0.01314 | 0.00272 | -0.09289 | 0.003321 | -0.13096 |
| 13000 | -0.00041 | 0.009361 | -0.00023 | -0.06519 | -0.00162 | -0.14493 |
| 13200 | 0.001638 | -0.03308 | -6.2E-05 | -0.04644 | 0.002689 | -0.09038 |
| 13400 | 0.000253 | -0.01358 | 0.000115 | 0.070468 | 0.000317 | 0.008246 |
| 13600 | -0.00076 | 0.009502 | -0.00177 | 0.001157 | -0.00218 | -0.04734 |
| 13800 | 0.000051 | 0.004163 | -2.5E-05 | -0.08224 | -0.00039 | -0.0899 |
| 14000 | 0.001468 | -0.00926 | 0.00201 | -0.15184 | 0.002138 | -0.16563 |
| 14200 | 0.000142 | -0.05214 | 0.000835 | -0.22375 | 0.000319 | -0.33494 |
| 14400 | 0.000586 | -0.01499 | 0.000326 | -0.18851 | 0.001368 | -0.25394 |
| 14600 | 0.000346 | -0.0348 | -0.00015 | -0.12355 | -0.00071 | -0.18441 |
| 14800 | 0.000813 | 0.009997 | 0.000755 | -0.07908 | 0.000913 | -0.13199 |
| 15000 | -6.9E-05 | -0.03622 | -0.0002 | -0.12552 | -0.00141 | -0.17453 |
| 15200 | -0.00047 | -0.02313 | -0.00065 | -0.12069 | -0.00025 | -0.18441 |
| 15400 | 0.00364 | 0.052051 | 0.002124 | -0.07995 | -0.00069 | -0.13398 |
| 15600 | 0.002494 | 0.005731 | -0.00023 | -0.09986 | 0.000636 | -0.13771 |
| 15800 | 0.000086 | 0.008433 | 0.000189 | -0.10474 | 0.000181 | -0.14497 |
| 16000 | 0.001159 | 0.008574 | 0.000048 | -0.07967 | -0.00071 | -0.13454 |
| 16200 | 0.000165 | 0.013913 | 0.000633 | -0.06175 | 0.00043 | -0.11853 |
| 16400 | -0.00085 | -0.00363 | -0.00066 | -0.08137 | 0.000003 | -0.12465 |
| 16600 | 0.000837 | 0.006764 | -0.00109 | -0.06468 | -2.1E-05 | -0.09356 |
| 16800 | -0.00051 | -0.0196 | -0.0001 | -0.06525 | -0.0012 | -0.09714 |
| 17000 | -0.00052 | 0.004163 | -0.00067 | -0.04469 | 0.002295 | -0.06393 |
| 17200 | -0.00194 | -0.00147 | -0.00059 | -0.04644 | 0.000202 | -0.07427 |
| 17400 | -0.00084 | -0.01837 | 0.000215 | -0.10389 | -0.00125 | -0.13506 |
| 17600 | -0.00044 | -0.00652 | -0.00012 | -0.09382 | 0.001296 | -0.13557 |
| 17800 | -2.2E-05 | 0.025828 | 0.002714 | -0.04763 | 0.001476 | -0.09093 |
| 18000 | -0.00134 | 0.033374 | 0.000016 | -0.05743 | 0.001168 | -0.07321 |
| 18200 | 0.001569 | 0.002843 | 0.000237 | -0.07152 | 0.001091 | -0.06181 |
| 18400 | 0.000017 | 0.003235 | 0.001278 | -0.04845 | 0.003072 | -0.06027 |
| 18600 | 0.00017 | -0.02102 | 0.001048 | -0.03461 | -0.00041 | -0.08576 |
| 18800 | 0.000519 | -0.01157 | 0.000037 | -0.05946 | 0.000639 | -0.08203 |
| 19000 | -0.00174 | -0.01313 | -0.00099 | -0.05692 | -0.00234 | -0.08728 |
| 19200 | 0.00071 | 0.002602 | 0.000658 | -0.04362 | -0.00082 | -0.05396 |
| 19400 | -0.00073 | -0.01127 | -0.00096 | -0.04068 | -0.00014 | -0.02645 |
| 19600 | 0.000215 | 0.010392 | -0.00035 | -0.02075 | -0.00034 | -0.03122 |
| 19800 | 0.001193 | 0.005734 | 0.003119 | 0.006635 | 0.002371 | -0.0229 |
| 20000 | 0.000217 | -0.02309 | -0.00045 | -0.02742 | 0.000094 | -0.01251 |
| 20200 | -0.00081 | -0.02063 | -0.00152 | -0.06632 | 0.0002 | -0.04521 |
| 20400 | -0.00308 | -0.03195 | -0.00077 | -0.05884 | -0.00235 | -0.07328 |
| 20600 | -0.00081 | -0.0374 | -0.00153 | -0.10243 | -0.00155 | -0.08778 |
| 20800 | 0.00146 | -0.03102 | 0.000998 | -0.07332 | 0.001092 | -0.09303 |
| 21000 | -0.00061 | -0.01118 | -0.00163 | -0.0814 | -0.00089 | -0.08571 |
| 21200 | -0.00126 | -0.04205 | 0.000441 | -0.08024 | 0.000451 | -0.08047 |
| 21400 | -0.00118 | 0.001568 | -0.00051 | -0.07792 | -1.7E-05 | -0.09716 |
| 21600 | 0.000022 | -0.02583 | 0.000244 | -0.06866 | 0.000252 | -0.12001 |
| 21800 | 0.002172 | 0.004022 | 0.000734 | -0.0649 | 0.001162 | -0.10437 |
| 22000 | 0.000728 | -0.00299 | -0.00123 | -0.10536 | -0.00027 | -0.14755 |
| 22200 | 0.000665 | 0.00142 | -0.00063 | -0.10796 | 0.000385 | -0.12417 |
| 22400 | 0.000728 | 0.018966 | 0.000859 | -0.0528 | 0.00054 | -0.07896 |
| 22600 | 0.00038 | -0.00142 | -0.00039 | -0.08919 | 0.000159 | -0.13242 |
| 22800 | -0.00312 | 0.022591 | -0.0006 | -0.03639 | -0.00184 | -0.09764 |
| 23000 | -0.00164 | 0.038368 | -0.00091 | -0.00773 | -0.00146 | -0.03694 |
| 23200 | -6E-06 | 0.083016 | 0.001111 | 0.004688 | 0.001432 | -0.00328 |
| 23400 | -0.00062 | 0.058811 | 0.000456 | -0.05912 | 0.00116 | -0.08844 |
| 23600 | 0.000388 | -0.01103 | 0.000717 | -0.14927 | 0.002276 | -0.2057 |
| 23800 | 0.000517 | -0.02744 | 0.001141 | -0.15246 | 0.000527 | -0.23208 |
| 24000 | -0.001 | -0.04729 | -0.00058 | -0.15477 | -0.00092 | -0.24663 |
| 24200 | 0.000552 | -0.01044 | 0.000723 | -0.09153 | 0.000817 | -0.14276 |
| 24400 | -0.00387 | -0.05195 | -0.00031 | -0.08399 | -0.00232 | -0.12157 |
| 24600 | -0.0009 | -0.02127 | -0.00042 | -0.03574 | 0.000092 | -0.07222 |
| 24800 | 0.001455 | -0.00716 | 0.000821 | -0.0367 | 0.000935 | -0.08105 |
| 25000 | 0.000908 | -0.01196 | 0.002258 | -0.03668 | -0.00027 | -0.09038 |
| 25200 | 0.002563 | -0.00235 | 0.000176 | -0.02134 | 0.000681 | -0.07018 |
| 25400 | -0.00207 | -0.00858 | 0.00043 | -0.05223 | 0.00055 | -0.10185 |
| 25600 | 0.001507 | 0.003088 | 0.000291 | -0.06779 | -0.0006 | -0.11635 |
| 25800 | -0.00072 | -0.00838 | 0.000289 | -0.067 | -0.00103 | -0.08723 |
| 26000 | -0.00154 | -0.0053 | -0.0003 | -0.05457 | 0.001068 | -0.08309 |
| 26200 | -0.0015 | 0.012743 | -0.00153 | -0.04297 | -0.00169 | -0.09719 |
| 26400 | 0.000341 | 0.040136 | 0.00177 | -0.02134 | 0.000384 | -0.07434 |
| 26600 | 0.000876 | 0.026264 | 0.000608 | -0.05048 | 0.001072 | -0.08266 |
| 26800 | -0.00177 | 0.044494 | 0.00024 | -0.02473 | 0.000453 | -0.07021 |
| 27000 | -0.00091 | 0.057184 | -0.00037 | -0.02764 | 0.000047 | -0.07545 |
| 27200 | 0.000528 | -0.00054 | 0.000241 | -0.08543 | -2.1E-05 | -0.12513 |
| 27400 | -0.00078 | -0.02171 | 0.000049 | -0.09263 | -0.00069 | -0.14175 |
| 27600 | -0.00029 | -0.01921 | 0.000492 | -0.07103 | 0.000343 | -0.11471 |
| 27800 | -0.00067 | -0.01529 | 0.001468 | -0.07792 | 0.000977 | -0.12266 |
| 28000 | -0.00058 | -0.00064 | 0.000303 | -0.07211 | -0.00107 | -0.08932 |
| 28200 | -0.00161 | -0.00559 | -0.00049 | -0.06172 | -0.00248 | -0.09714 |
| 28400 | -0.00388 | 0.03259 | -0.00092 | -0.0179 | -0.00037 | -0.05349 |
| 28600 | -0.00201 | 0.003526 | -0.00038 | -0.04416 | -0.00146 | -0.05926 |
| 28800 | 0.001234 | 0.014697 | 0.00124 | -0.03405 | 0.002303 | -0.05465 |
| 29000 | -0.00322 | -0.0101 | 0.00125 | -0.03174 | -0.00078 | -0.07535 |
| 29200 | 0.000036 | -0.01 | 0.000088 | -0.06378 | -0.00021 | -0.0982 |
| 29400 | -0.0001 | -0.00676 | -0.00035 | -0.06409 | -0.00048 | -0.09456 |
| 29600 | 0.000577 | 0.015932 | 0.000807 | -0.06008 | 0.001591 | -0.09081 |
| 29800 | -0.0009 | 0.005048 | -0.00014 | -0.05573 | 0.000674 | -0.0612 |
| 30000 | 0.000051 | 0.015092 | -0.00011 | 0.015332 | -0.00046 | -0.01097 |
| 30200 | 0.00124 | 0.000539 | 0.000428 | -0.00745 | 0.001299 | -0.01712 |
| 30400 | 0.000622 | 0.005981 | 0.001924 | -0.01073 | 0.001093 | -0.01707 |
| 30600 | 0.00005 | 0.010098 | -0.00165 | -0.02541 | -0.00037 | -0.0701 |
| 30800 | 0.000631 | -0.00314 | 0.000518 | -0.07044 | 0.000708 | -0.10491 |
| 31000 | -0.00098 | -0.00559 | -0.00217 | -0.08659 | -0.00239 | -0.13714 |
| 31200 | 0.001039 | 0.00505 | 0.001806 | -0.08749 | 0.001774 | -0.11837 |
| 31400 | -0.00196 | 0.006226 | -0.00058 | -0.04941 | -0.00168 | -0.12311 |
| 31600 | -0.00233 | -0.00299 | -0.00085 | -0.05827 | -0.00111 | -0.10284 |
| 31800 | -0.00026 | -0.02284 | 0.000938 | -0.0799 | -0.00068 | -0.12422 |
| 32000 | 0.001159 | 0.014019 | 0.001226 | -0.05048 | 0.002209 | -0.08049 |
| 32200 | 0.001114 | 0.042196 | 0.000478 | -0.01586 | 0.001337 | -0.05558 |
| 32400 | -0.00098 | 0.000928 | 0.001631 | -0.04269 | -0.00118 | -0.06587 |
| 32600 | -0.00208 | -0.00412 | 0.000366 | -0.03664 | 0.000453 | -0.07227 |
| 32800 | 0.001704 | 0.046362 | 0.002449 | -0.02428 | 0.000754 | -0.07432 |
| 33000 | -0.00039 | 0.019213 | 0.001188 | -0.0563 | 0.000615 | -0.10697 |
| 33200 | -0.0005 | -0.02573 | -0.00128 | -0.12036 | -0.00157 | -0.19533 |
| 33400 | -0.00236 | 0.001175 | -0.0019 | -0.10537 | -0.00228 | -0.17564 |
| 33600 | 0.000517 | 0.002207 | 0.001114 | -0.10186 | 0.001704 | -0.14492 |
| 33800 | 0.000144 | 0.034305 | -0.00077 | -0.05887 | 0.000728 | -0.11378 |
| 34000 | 0.00009 | 0.049105 | -0.00034 | -0.05599 | -0.00077 | -0.10755 |
| 34200 | 0.0025 | 0.016759 | 0.001303 | -0.04619 | 0.001072 | -0.0816 |
| 34400 | 0.000468 | -0.04323 | 0.000708 | -0.06928 | 0.000247 | -0.12937 |
| 34600 | -0.00116 | -0.00961 | -0.00068 | -0.04907 | -6.5E-05 | -0.07008 |
| 34800 | 0.000399 | 0.013233 | 0.000592 | -0.04616 | 0.000749 | -0.06751 |
| 35000 | 0.000431 | 0.003236 | 0.000582 | -0.05082 | 0.000041 | -0.07111 |
| 35200 | -0.00171 | 0.023377 | -0.00053 | -0.00754 | -0.00148 | -0.04469 |
| 35400 | 0.000506 | -0.00559 | 0.000492 | -0.05856 | -0.00064 | -0.05762 |
| 35600 | 0.000079 | 0.025434 | 0.000481 | -0.03894 | 0.000637 | -0.05407 |
| 35800 | -0.00075 | -0.00715 | -0.00187 | -0.06638 | 0.000544 | -0.06342 |
| 36000 | -0.00137 | -0.03597 | 0.000669 | -0.06146 | 0.001523 | -0.08362 |
| 36200 | -0.00147 | 0.018032 | -0.00163 | -0.02536 | -0.00316 | -0.03274 |
| 36400 | -0.00189 | 0.019258 | -0.00163 | 0.023436 | -0.00237 | 0.017049 |
| 36600 | -0.00017 | 0.013421 | -0.00097 | 0.125887 | 0.000388 | 0.117865 |
| 36800 | -0.00052 | 0.001755 | -0.00076 | 0.176987 | -0.00114 | 0.188446 |
| 37000 | -0.00088 | -0.01765 | -0.00015 | 0.082015 | -0.00052 | 0.098548 |
| 37200 | -0.00165 | -0.05582 | -0.00013 | -0.04729 | -0.0007 | -0.0603 |
| 37400 | -0.00081 | -0.04479 | -0.00063 | -0.10243 | -0.00093 | -0.1226 |
| 37600 | -0.00011 | -0.05033 | -0.00138 | -0.2742 | -0.0007 | -0.34596 |
| 37800 | 0.000603 | -0.0051 | 0.000844 | -0.20031 | 0.000837 | -0.31373 |
| 38000 | -0.00067 | 0.066256 | -0.00033 | -0.02101 | 0.000274 | -0.0613 |
| 38200 | 0.00079 | 0.067823 | 0.000633 | 0.056888 | 0.000907 | 0.069069 |
| 38400 | 0.000858 | 0.055573 | 0.00192 | 0.002031 | 0.000815 | 0.010842 |
| 38600 | 0.001236 | 0.046754 | 0.001051 | -0.02744 | -1.9E-05 | -0.03946 |
| 38800 | 0.001743 | 0.062046 | 0.000821 | 0.025608 | 0.001092 | -0.01034 |
| 39000 | -0.00145 | 0.073219 | -0.00169 | 0.025098 | -0.00164 | 0.024459 |
| 39200 | 0.000979 | 0.079881 | 0.000164 | 0.031508 | 0.000933 | 0.037901 |
| 39400 | 0.002599 | 0.021709 | 0.001782 | -0.00836 | 0.001164 | -0.02809 |
| 39600 | 0.001507 | 0.026223 | -0.00021 | -0.04331 | 0.000431 | -0.04466 |
| 39800 | 0.001599 | 0.003238 | 0.000846 | -0.08718 | 0.000505 | -0.09968 |
| 40000 | 0.001777 | -0.00676 | -0.00029 | -0.06031 | -0.00041 | -0.07167 |
| 40200 | -0.00058 | -0.04195 | 0.000356 | -0.07529 | 0.000112 | -0.06236 |
| 40400 | 0.000022 | -0.03989 | 0.00033 | -0.14602 | -0.0008 | -0.11113 |
| 40600 | 0.001159 | -0.08405 | 0.00015 | -0.22456 | 0.000114 | -0.23266 |
| 40800 | -0.00141 | -0.07228 | -0.00149 | -0.27281 | -0.00308 | -0.29918 |
| 41000 | -0.00053 | 0.00579 | -0.00039 | -0.24684 | -0.00014 | -0.256 |
| 41200 | -0.00048 | 0.094783 | 0.000567 | -0.01598 | 0.000569 | -0.01856 |
| 41360 | -0.00246 | 0.313938 | -0.0008 | 0.510472 | -0.00068 | 0.550244 |

**Table S5.** Raw data of the Turbiscan Stability Index (TSI) measurements of extemporaneous suspensions of hydroxychloroquine sulphate, after tablet manipulation, in fast oral solution “Puccini” and fast oral solution - sugar free “Beethoven” depicted in Fig. S2 (panel A and B).

| Fast oral solution  “Puccini” | | Fast oral solution - sugar free “Beethoven” | |
| --- | --- | --- | --- |
| Time (s) | TSI | Time (s) | TSI |
| 0 | 0 | 0 | 0 |
| 25 | 0.378359 | 1800 | 0,015433 |
| 50 | 0.750125 | 3600 | 0,031481 |
| 75 | 1.127371 | 5400 | 0,047913 |
| 100 | 1.504535 | 7200 | 0,0645 |
| 125 | 1.892955 | 9000 | 0,081008 |
| 150 | 2.303267 | 10800 | 0,097211 |
| 175 | 2.722328 | 12600 | 0,112874 |
| 200 | 3.136705 | 14400 | 0,127769 |
| 225 | 3.507609 | 16200 | 0,141666 |
| 250 | 3.835239 | 18000 | 0,15433 |
| 275 | 4.131399 | 19800 | 0,165537 |
| 300 | 4.404082 | 21600 | 0,175245 |
| 325 | 4.672245 | 23400 | 0,183689 |
| 350 | 4.939586 | 25200 | 0,191032 |
| 375 | 5.220714 | 27000 | 0,19744 |
| 400 | 5.504732 | 28800 | 0,203077 |
| 425 | 5.776035 | 30600 | 0,208108 |
| 450 | 6.030491 | 32400 | 0,212699 |
| 475 | 6.249275 | 34200 | 0,217015 |
| 500 | 6.435439 | 36000 | 0,22122 |
| 525 | 6.593843 | 37800 | 0,225481 |
| 550 | 6.734286 | 39600 | 0,229962 |
| 575 | 6.859616 | 41400 | 0,234828 |
| 600 | 6.978368 | 43200 | 0,240245 |
| 625 | 7.093702 |  |  |
| 650 | 7.214368 |  |  |
| 675 | 7.33538 |  |  |
| 700 | 7.454011 |  |  |
| 725 | 7.567578 |  |  |
| 750 | 7.679135 |  |  |
| 775 | 7.786257 |  |  |
| 800 | 7.891829 |  |  |
| 825 | 7.993583 |  |  |
| 850 | 8.094227 |  |  |
| 875 | 8.191772 |  |  |
| 900 | 8.288425 |  |  |
| 925 | 8.383118 |  |  |
| 950 | 8.474124 |  |  |
| 975 | 8.564263 |  |  |
| 1000 | 8.651073 |  |  |
| 1025 | 8.736359 |  |  |
| 1050 | 8.818062 |  |  |
| 1075 | 8.897997 |  |  |
| 1100 | 8.974074 |  |  |
| 1125 | 9.048637 |  |  |
| 1150 | 9.120303 |  |  |
| 1175 | 9.190685 |  |  |
| 1200 | 9.258121 |  |  |
| 1225 | 9.324206 |  |  |
| 1250 | 9.387374 |  |  |
| 1275 | 9.448683 |  |  |
| 1300 | 9.508117 |  |  |
| 1325 | 9.56455 |  |  |
| 1350 | 9.619326 |  |  |
| 1375 | 9.671148 |  |  |
| 1400 | 9.720928 |  |  |
| 1425 | 9.768702 |  |  |
| 1450 | 9.813907 |  |  |
| 1475 | 9.857775 |  |  |
| 1500 | 9.899887 |  |  |
| 1525 | 9.939617 |  |  |
| 1550 | 9.978413 |  |  |
| 1575 | 10.0157 |  |  |
| 1600 | 10.05114 |  |  |
| 1625 | 10.08582 |  |  |
| 1650 | 10.11892 |  |  |
| 1675 | 10.15137 |  |  |
| 1700 | 10.18246 |  |  |
| 1725 | 10.21307 |  |  |
| 1750 | 10.24256 |  |  |
| 1775 | 10.27173 |  |  |
| 1800 | 10.29997 |  |  |
| 1825 | 10.3281 |  |  |
| 1850 | 10.35588 |  |  |
| 1875 | 10.383 |  |  |
| 1900 | 10.41011 |  |  |
| 1925 | 10.43618 |  |  |
| 1950 | 10.46189 |  |  |
| 1975 | 10.48692 |  |  |
| 2000 | 10.51096 |  |  |
| 2025 | 10.53475 |  |  |
| 2050 | 10.55764 |  |  |
| 2075 | 10.58021 |  |  |
| 2100 | 10.60194 |  |  |
| 2125 | 10.62338 |  |  |
| 2150 | 10.64426 |  |  |
| 2175 | 10.66427 |  |  |
| 2200 | 10.6841 |  |  |
| 2225 | 10.70343 |  |  |
| 2250 | 10.72201 |  |  |
| 2275 | 10.74042 |  |  |
| 2300 | 10.75816 |  |  |
| 2325 | 10.77567 |  |  |
| 2350 | 10.79279 |  |  |
| 2375 | 10.80922 |  |  |
| 2400 | 10.82555 |  |  |
| 2425 | 10.84133 |  |  |
| 2450 | 10.85694 |  |  |
| 2475 | 10.87222 |  |  |
| 2500 | 10.88697 |  |  |
| 2525 | 10.90165 |  |  |
| 2550 | 10.91587 |  |  |
| 2575 | 10.92986 |  |  |
| 2600 | 10.9432 |  |  |
| 2625 | 10.95618 |  |  |
| 2650 | 10.96869 |  |  |
| 2675 | 10.98055 |  |  |
| 2700 | 10.99216 |  |  |
| 2725 | 11.00336 |  |  |
| 2750 | 11.01398 |  |  |
| 2775 | 11.0244 |  |  |
| 2800 | 11.03445 |  |  |
| 2825 | 11.04402 |  |  |
| 2850 | 11.05341 |  |  |
| 2875 | 11.0625 |  |  |
| 2900 | 11.07117 |  |  |
| 2925 | 11.07971 |  |  |
| 2950 | 11.08791 |  |  |
| 2975 | 11.09598 |  |  |
| 3000 | 11.10374 |  |  |
| 3025 | 11.11141 |  |  |
| 3050 | 11.11882 |  |  |
| 3075 | 11.12616 |  |  |
| 3100 | 11.13336 |  |  |
| 3125 | 11.14035 |  |  |
| 3150 | 11.14736 |  |  |
| 3175 | 11.1542 |  |  |
| 3200 | 11.16107 |  |  |
| 3225 | 11.16782 |  |  |
| 3250 | 11.17463 |  |  |
| 3275 | 11.18137 |  |  |
| 3300 | 11.1882 |  |  |
| 3325 | 11.19499 |  |  |
| 3350 | 11.20191 |  |  |
| 3375 | 11.20892 |  |  |
| 3400 | 11.2159 |  |  |
| 3425 | 11.22311 |  |  |
| 3450 | 11.23036 |  |  |
| 3475 | 11.23785 |  |  |
| 3500 | 11.24552 |  |  |
| 3525 | 11.25326 |  |  |
| 3550 | 11.26133 |  |  |
| 3575 | 11.26952 |  |  |
| 3600 | 11.27806 |  |  |
